# Effect of basal luteinizing hormone (bLH) level on in vitro fertilization/intra-cytoplasmic injections (IVF/ICSI) outcomes in polycystic ovarian syndrome (PCOS) patients

**DOI:** 10.1186/s12884-023-05944-4

**Published:** 2023-08-29

**Authors:** Zhuo Liu, Ke-Hua Wang

**Affiliations:** 1 The First Clinical College, Shandong University of Traditional Chinese Medicine, Jinan, Shandong China; 2 Reproduction and Genetics Center, Affiliated Hospital of Shandong University of Traditional Chinese Medicine, Jinan, Shandong China

**Keywords:** Polycystic ovary syndrome (PCOS), Oral contraceptive, Luteinizing hormone (LH), *In vitro* fertilization and embryo transfer, Clinical pregnancy outcome

## Abstract

**Objective:**

To evaluate the effect of basal luteinizing hormone (bLH) levels on In Vitro Fertilization/Intra-Cytoplasmic Injections (IVF/ICSI) outcomes in polycystic ovary syndrome (PCOS).

**Methods:**

A total of 256 PCOS patients who underwent IVF/ICSI treatment in our center from January 2018 to January 2022 were analyzed retrospectively. The patients were based on the third quartile (12.455) of the basal LH value was taken as the cut-off value and was divided into high and low LH group: high LH group (LH ≥ 12.455 IU / L) and low LH group (LH < 12.455 IU / L) and the OC group was pretreated with oral contraceptives. The outcomes in ovulation induction and embryo transfer cycles of the three groups were then compared. In addition, factors influencing the number of good quality embryos and the early onset LH peak were analyzed.

**Results:**

Ages, infertility duration, body mass index (BMI), and basal follicle-stimulating hormone (FSH), and progesterone (P), testosterone (T) levels were not significantly different among the three groups (*p* > 0.05). However,there were significant differences in basal LH and basal E2 between low LH group and high LH group, and there were significant differences in basal LH between high LH group and OC group (*p* < 0.05). LH on the antagonist day was significantly different between low LH group and high LH group and between high LH group and OC group (*p* < 0.05). LH on the hCG (human Chorionic Gonadotropin) day there were significant differences between low LH group and OC group, high LH group and OC group (*p* < 0.05). The Mode of triggering between the three groups had significant differences between the two groups (*p* < 0.05). In addition, the number of days from gonadotropin (Gn) initiation to antagonist addition were significantly different among the three groups (*p* < 0.05). In addition, total Gn doses,the number of oocytes retrieved, the number of Gn days, 2pronucleus (2PN) numbers, number of good quality embryos, and number of high risk OHSS (Ovarian Hyper-stimulation Syndrome), cases with OHSS occurrences were not significantly different among the three groups (*p* > 0.05). Moreover, the cycle and clinical pregnancy outcomes and the cumulative clinical pregnancy rate and the cumulative live birth rate were not significantly different among the three groups (*p* > 0.05). LH levels on the day of antagonist addition affected the number of good-quality embryos (B < 0, *p* < 0.05). However, LH levels on the day antagonist was added were not significantly correlated with basal LH levels (Pearson correlation coefficient = 0.259), the ROC curve was constructed for the logistic prediction model of the early onset LH peak, and the AUC value was 0.747, indicating that the logistic combined model we constructed had a good ability to predict the early onset LH peak.

**Conclusion:**

Basal LH levels do not affect the pregnancy outcomes in PCOS patients after antagonist protocols. Besides, LH levels on the day of antagonist addition affect the number of good quality embryos for PCOS patients undergoing IVF /ICSI.

## Introduction

PCOS is clinically characterized by hyperandrogenism, ovulatory failure, and polycystic ovary morphology [1]. Its endocrine profile is characterized by increased levels of luteinizing hormone and testosterone. The incidence of PCOS in women of reproductive age and those with ovulatory infertility is about 4-20% and 70%, respectively [2]. Although there are several PCOS-related studies, the pathogenic mechanisms of PCOS are unclear [3, 4]. Serum LH levels are upregulated in PCOS patients and maintained at normal or low FSH levels, thus increasing the ratio of LH and FSH (LH/FSH), leading to the impairment of follicular maturation and infertility [5–7]. This is caused by the changes in the specific pattern of gonadotropin-releasing hormone (GnRH) secretion in patients, abnormalities in the negative feedback mechanism of oestrogens and progesterone, and multifactorial influences, such as hyperandrogenism, hyperinsulinaemia, and obesity. High basal LH levels can induce theca cells, promote testosterone secretion, and affect follicle development, thus decreasing oocyte quality and increasing the risk of miscarriage [8]. As a result, oral contraceptives (OC) are usually routinely prescribed to women with PCOS for 3–6 months before they enter ovulatory promoting cycle to downregulate LH and T levels in the body, improving the efficacy of ovulatory treatment, and reduce miscarriage rates [9, 10]. However, it is currently controversial whether PCOS patients should take OC before IVF/ICSI treatment. Moreover, it is unclear whether elevated basal LH levels can impact the ovulatory effect and clinical outcome [11]. Studies have found [12, 13] that the rate of spontaneous pregnancy within 3 months after stopping taking OC is significantly lower than that of the non-OC group, indicating that the effect of OC on pregnancy is persistent [14, 15].

Early-onset LH peak in antagonist protocols affects oocyte, embryo quality, and clinical pregnancy outcomes [16–18]. However, a transient abnormal increase in LH during the surge cannot affect the clinical outcome [19]. Furthermore, it is unclear whether basal LH levels affect pregnancy outcomes in PCOS patients. In this study, the effect of basal LH rise on ovulation induction and pregnancy outcomes in PCOS patients was retrospectively examined using antagonist protocols.

## Materials and methods

### Study subjects

**Fig. 1:**
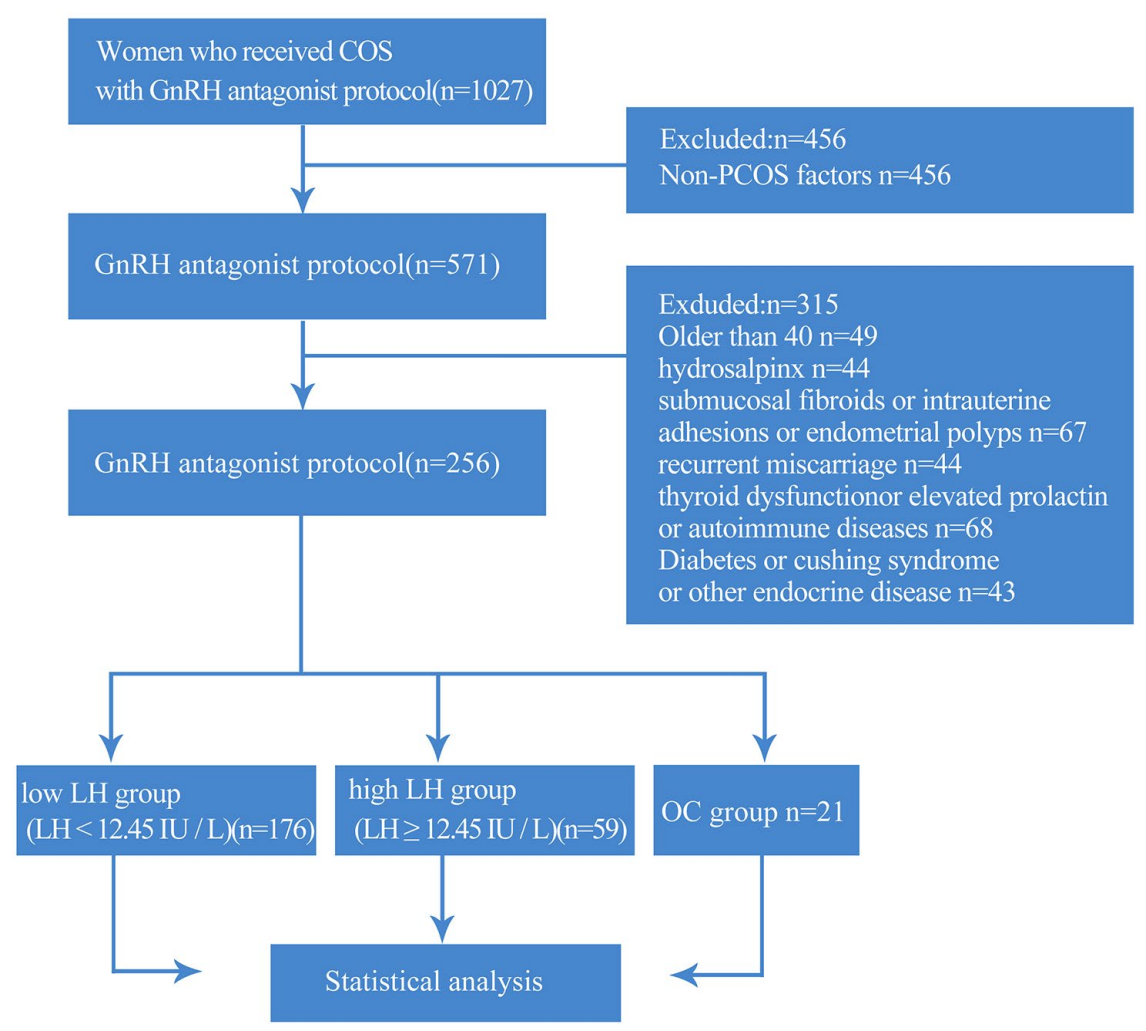
Case inclusion and exclusion criteria

This study included 256 PCOS patients who in undergoing IVF/ICSI cycles at the reproductive center of Shandong University of Traditional Chinese medicine from January 2018 to January 2022 (Fig. 1). All processes of this study were approved by the hospital Ethics Committee. PCOS diagnostic criteria followed those recommended by the Rotterdam expert meeting held by ESHRE/ASRM 2003 (the European Society of Human Reproduction and Embryology,the American Society for Reproductive Medicine) [20].

Inclusion criteria:

1. Patients aged 20–40 years;
2. Patients who had fulfilled the diagnostic criteria for PCOS;
3. Patients who underwent IVF/ICSI treated and the men’s semen showed no abnormality;

Exclusion criteria:

1. Patients with bilateral or unilateral hydrosalpinx detected by B-ultrasound or hysterosalpingography;
2. Patients with submucosal fibroids or intrauterine adhesions, endometrial polyps, etc.;
3. Patients with a history of recurrent miscarriage;
4. Patients with thyroid dysfunction, elevated prolactin and autoimmune diseases;
5. Patients with diabetes, cushing syndrome, and other endocrine disease;
6. congenital or acquired uterine anomalies, history of ovarian surgery;
7. abnormal parental karyotypes or medical conditions that contraindicated assisted reproductive technology and/or pregnancy;
8. previous medication of combined oral contraceptive pills or glucocorticosteroids within 2–3 months before ovarian stimulation;
9. repeated cycles, one patient with more than one time of COH.

### Ovulation induction protocol

The enrolled patients were put on a flexible antagonist protocol.patients on menstrual day 2 or 3. B-ultrasound excluded abnormalities, such as bilateral ovarian cysts. FSH, LH, E2, P, T and PRL levels were also measured. The starting Gn dose (only Gonafine, Merck Serono,Switzerland) was based on patient age, BMI, AFC (Antral Follicle Coun) levels. The patients returned to the hospital 4–5 days after the first dose and every 2–3 days to monitor B-ultrasound and LH, E2, P. The Gn dose was adjusted based on ovarian responsiveness. Antagonist (MerckSerono,Switzerland) was subcutaneously administered (0.25 mg/d) when the dominant follicle diameter was between 12 and 14 mm and E2 ≥ 300pg/L until trigger day. LH levels were monitored every 24–72 h depending on follicle size. An antagonist (0.5 mg/d) was administrated the same day for an early onset LH peak (LH ≥ 12.455 U/L).The LH and P levels were then monitored every 24–48 h before subcutaneous injection with 0.25 mg/d antagonist. Chorionic gonadotrophin (Merck Serono, Switzerland) or 0.1–0.2 mg subcutaneous dabigatran (Huiling, Switzerland) was administered when two and more follicles had a diameter ≥ 18 mm or when three and more follicles had a diameter of ≥ 17 mm; after 36-38 h by B-ultrasound assisted in oocyte retrieval. Embryo was transferred after 3–5 days of culture in vitro. The patients for fresh embryo transplantation received luteal support on the day of oocyte retrieval. The patients did not meet the conditions of fresh embryo transfer when the number of retrieved oocytes was ≥ 15, or they had a tendency of ovarian hyperstimulation, such as abdominal distension and ascites, and when progesterone was upregulated in advance (≥ 2ng/l). All embryos from such patients was frozen.

### In vitro fertilization and embryo quality evaluation

IVF or ICSI fertilization was selected after oocyte retrieval depending on sperm condition or previous history of fertilization. The quality of the day 3 (D3) embryo was scored by evaluating the specific number, size, shape, symmetry, proportion of fragments in an embryo, and the thickness of zona pellucida. High-quality embryos were those in grade II and above (Grade I:the size of blastomere is uniform, and the cytoplasm is no different, fragments < 5%; Grade II:the size of the blastomere is uniform or uneven, broken pieces < 20%). The D3 embryos (1–2) were transferred after oocyte retrieval, and the untransmitted embryos were cultured until the D5 blastocysts were frozen.

### Embryo transfer and luteal support

Patients with OHSS risk were treated with GnRH-a(gonadotropin releasing hormone agomist) single trigger, and all embryos were cryopreserved. Oral dydrogesterone tablets (Duff,Abbott Biologicals, the Netherlands), 10 mg bid, were given daily after oocyte retrieval in patients with GnRH-a plus low dose hCG dual trigger or hCG trigger.at the same time,progesterone injection (Zhejiang Xianju)40 mg/d intramuscular injection or vaginal progesterone sustained release gel (Merck Serono) 90 mg was used; Serum hCG levels were measured 14 days after embryo transfer. B-ultrasound examination confirmed clinical pregnancy after 35 d of embryo transfer.

### Statistical analysis

The basal LH grouping was performed by the quartile method. Enumeration data were expressed as frequency. The chi-square test with the Bonferroni method was used to comparatively analyze the data between the two groups. The Shapiro-Wilk test was used to assess the normality of measurement data. Data with normal distribution and homogeneity of variance were expressed as mean ± standard deviation (SD). The data were comparatively analyzed between groups using the ANOVA analysis. Otherwise, the Kruskal-Wallis rank sum test was used for comparison between the two groups. LSD test or Bonfferoni correction for pairwise comparisons. Difference analysis was used to explore the clinical indicators related to LH value, and a multivariate logistic regression model was used to jointly predict the LH level of multiple variables. All statistical tests were two-sided (α = 0.05) and were performed using SPSS 26.0. *p* < 0.05 was considered statistically significant.

## Results

### Effect of basal LH level on the outcome of IVF/ICSI in PCOS patients

The patients were based on the third quartile (12.455) of the basal LH value was taken as the cut-off value and was divided into high LH group (59 patients) (LH ≥ 12.455 IU/L) and low LH group (176 patients) (LH < 12.455 IU/L), and other group is Oral contraceptive group (OC group) (n = 21).

### Comparison of clinical data

The ages, infertility duration, BMI, basal FSH, T and P levels were not significantly different among the three groups **(**Table 1**)** (*p* > 0.05). However,there were significant differences in basal LH and basal E2 between low LH group and high LH group, and there were significant differences in basal LH between high LH group and OC group (*p* < 0.05).

**Table 1:** Clinical data of patients among the three groups

Index	Low LH group (n = 176)	High LH group (n = 59)	OC group (n = 21)	*p* value
Age	30.63 ± 4.16	30.41 ± 4.26	31.52 ± 2.56	0.555
Durationof infertility (years)	3.58 ± 2.48	3.93 ± 2.79	3.76 ± 2.26	0.649
BMI (kg/m2)	26.12 ± 4.35	24.55 ± 4.12	25.76 ± 3.46	0.050
BasalFSH (IU/L)	6.64 ± 1.50	7.04 ± 1.45	6.51 ± 2.12	0.188
BasalLH (IU/L)	7.53 ± 2.34a	17.16 ± 4.44a,b	6.22 ± 2.28b	< 0.001
BasalE2 (pg/L)	44.19 ± 20.49a	52.7 ± 22.06a	40.25 ± 13.49	0.007
BasalP (ng/mL)	0.68 ± 0.68	0.79 ± 1.08	0.63 ± 0.41	0.815
BasalT (ng/mL)	0.63 ± 0.53	0.65 ± 0.29	0.70 ± 0.37	0.130

a:*p* < 0.05,Low LH group is significantly different from High LH group

b:*p* < 0.05, High LH group is significantly different from OC group

### **The outcomes of ovulation induction among the three groups**

LH on the antagonist day was significantly different between low LH group and high LH group and between high LH group and OC group (*p* < 0.05). LH on the hCG day there were significant differences between low LH group and OC group, high LH group and OC group (*p* < 0.05). The Mode of triggering between the three groups had significant difference between the two groups (*p* < 0.05). In addition, the number of days from Gn initiation to antagonist addition were significantly different among the three groups (*p* < 0.05). Total Gn doses,the number of oocytes retrieved, the number of Gn days, 2PN numbers, number of good quality embryos, P levels on the hCG day, number of high risk OHSS, cases with OHSS occurrences were not significantly different among the three groups (*p* > 0.05) (Table 2).

**Table 2:** Comparison of ovulation induction outcomes among three groups of patients

Index	Low LH group	High LH group	OC group	*p* value
	(n = 176)	(n = 59)	(n = 21)	
Gn duration (days)	10.02 ± 2.80	9.58 ± 1.73	10.38 ± 2.80	0.679
Total Gn doses (U)	2115.68 ± 860.18	1931.19 ± 651.31	2325.60 ± 745.96	0.051
Number of collected oocytes	18.77 ± 9.45	21.81 ± 11.97	17.05 ± 7.12	0.169
Number of 2PN	10.97 ± 6.45	14.24 ± 10.61	10.43 ± 4.58	0.231
Number of high-quality embryos	4.27 ± 2.54	4.44 ± 2.91	4.90 ± 2.70	0.610
LH on the antagonist day (IU/L)	6.65 ± 5.35a	10.55 ± 11.57a,b	4.73 ± 2.98b	< 0.001
LH on the hCG day (IU/L)	3.79 ± 4.76c	3.90 ± 2.28b	2.18 ± 2.00b,c	0.002
Gn distance from antagonist days (days)	5.82 ± 1.93	5.2 ± 1.63	5.05 ± 1.16	0.017
The P grouping on the hCG day (ng/mL)				0.903
< 2	145(82.39)	48(81.36)	18(85.71)	
≥ 2	31(17.61)	11(18.64)	3(14.29)	
Mode of triggering				< 0.001
Single	144(81.82)a,c	38(64.41)a,b	2(9.52)b,c	
Combination	32(18.18)a,c	21(35.59)a,b	19(90.48)b,c	
Form of fertilization				0.480
IVF	159(90.34)	51(86.44)	20(95.24)	
ICSI	17(9.66)	8(13.56)	1(4.76)	
OHSS high risk				0.358
NO	73(41.48)	20(33.9)	6(28.57)	
YES	103(58.52)	39(66.1)	15(71.43)	
OHSS occurs				0.402
NO	174(98.86)	57(96.61)	21(100.00)	
YES	2(1.14)	2(3.39)	0(0.00)	

a:*P* < 0.05, Low LH group is significantly different from High LH group

b:*P* < 0.05, High LH group is significantly different from OC group

c:*P* < 0.05, Low LH group is significantly different from OC group

### **Pregnancy outcomes among the three groups**

The cycle outcome and clinical pregnancy outcome were not significantly different among the three groups (*p* > 0.05). Low LH group: A total of 176 cases, including 6 cases of no embryos, 36 cases of fresh embryo transfer, 17 cases of pregnancy, 134 cases of frozen embryo transfer, 114 cases of cumulative pregnancy, and 5 cases of remaining embryos without pregnancy, the cumulative clinical pregnancy rate was 17 + 114/165 = 79.39%, and the cumulative live birth rate was 99/165 = 60.00%.High LH group: A total of 59 cases, 3 cases of no embryos, 9 cases of fresh embryo transfer, 5 cases of pregnancy, and 47 cases of frozen embryo transfer, 39 cases of cumulative pregnancy, and 1 case of remaining embryos without pregnancy, the cumulative clinical pregnancy rate was 5 + 39/55 = 80.00%, the cumulative live birth rate was 34/55 = 61.82%.OC group: A total of 21 cases, including 1 case of no embryos, 4 cases of fresh embryo transfer, 2 cases of pregnancy, 16 cases of frozen embryo transfer, a total of 15 pregnancies, all embryos were transferred, the cumulative clinical pregnancy rate was 2 + 15/20 = 85.00%, the cumulative live birth rate was 11/20 = 55.00%. (Table 3).

**Table 3:** Comparison of clinical outcomes among the three groups

Index	Low LH group	High LH group	OC group	*p* value
Ovulation induction cycle outcome	(n = 176)	(n = 59)	(n = 21)	0.901
Whole-embryo freezing	134(76.14)	47(79.66)	16(76.19)	
Embryo transfer	36(20.45)	9(15.25)	4(19.05)	
No transplantable embryos	6(3.41)	3(5.08)	1(4.76)	
Outcome of fresh embryo pregnancy	(n = 36)	(n = 9)	(n = 4)	0.904
Pregnancy	17(47.22)	5(55.56)	2(50.00)	
Non-pregnancy	19(52.78)	4(44.44)	2(50.00)	
Outcome of frozen embryo pregnancy	(n = 134)	(n = 47)	(n = 16)	
				0.492
Pregnancy	71(52.99)	25(53.19)	11(68.75)	
				0.366
D3 embry pregnancy	54(40.30)	19(40.43)	8(50.00)	
				0.184
D5 embry pregnancy	17(12.69)	6(12.77)	3(18.75)	
	(n = 165)	(n = 55)	(n = 20)	
cumulative clinical pregnancy rate	131(79.39)	44(80.00)	17(85.00)	0.839
cumulative live birth rate	99(60.00)	34(61.82)	11(55.00)	0.868

### **Influencing factors of high-quality embryos in PCOS patients**

For multivariate linear regression analysis, the number of high-quality embryos was the dependent variable, while the number of Gn days, total Gn doses, duration of infertility, BMI, number of collected oocytes, basal levels of FSH, LH, E2, P, and T, numbers of 2PN, pregnancy outcome, LH, E2, and P levels on the day of hCG, and LH levels on the day of antagonist addition were independent variables (Table 4; R = 0.678, R2 = 0.460). The LH levels on the day of antagonist addition negatively affected the quality of embryos (B < 0, *p* < 0.05).

**Table 4:** Multivariate linear regression for the number of high-quality embryos in PCOS patients

Independent variable	B	Standard error	β	t	p	95% CI lower limit	95% CI upper limit
Gn duration (days)	-0.001	0.288	-0.002	-0.005	0.996	-0.593	0.590
Total Gn doses(U)	0.000	0.001	-0.083	-0.177	0.861	-0.002	0.002
Duration of infertility (years)	-0.191	0.141	-0.277	-1.349	0.189	-0.481	0.099
BMI(kg/m2)	0.085	0.077	0.202	1.109	0.277	-0.072	0.243
Number of collected oocytes	0.046	0.182	0.097	0.251	0.804	-0.328	0.420
Basal FSH(IU/L)	0.159	0.215	0.140	0.741	0.465	-0.282	0.601
Basal LH(IU/L)	0.142	0.071	0.342	2.003	0.055	-0.003	0.288
Basal E2(pg/L)	-0.016	0.016	-0.199	-1.008	0.323	-0.048	0.016
Basal P (ng/mL)	1.372	0.867	0.381	1.583	0.125	-0.406	3.151
Basal T (ng/mL)	-0.655	0.904	-0.156	-0.725	0.475	-2.509	1.199
Number of 2PN	0.042	0.218	0.076	0.191	0.850	-0.406	0.489
Pregnancy outcome	-1.100	0.773	-0.296	-1.423	0.166	-2.686	0.486
LH on the day of antagonist(IU/L)	-0.145	0.066	-0.404	-2.213	0.036	-0.280	-0.011
LH on the day of hCG(IU/L)	0.023	0.131	0.030	0.176	0.861	-0.245	0.291
E2 on the day of hCG(pg/L)	0.001	0.000	0.456	1.466	0.154	0.000	0.002
P on the day of hCG (ng/mL)	-0.156	0.464	-0.069	-0.336	0.739	-1.107	0.795
Timing of antagonist addition(days)	-0.310	0.244	-0.249	-1.272	0.214	-0.811	0.190

### **Prediction of risk of early onset LH peak in PCOS patients**

For binary logistic regression model, the early onset LH peak (LH ≥ 12.455) was the dependent variable, whereas the number of Gn days, total Gn doses, duration of infertility, BMI, number of collected oocytes, basal levels of FSH, LH, E2, P, and T, numbers of 2PN, number of high- quality embryos, number of days from Gn initiation to antagonist addition, E2 and P levels on the day of hCG were the independent variables (Table 5). The variables were screened using the enter method. The model had a good fitness (*p* = 0.787) based on Hosmer-Lemeshow test statistic (5.002). The result showed that the aforementioned variables were not the risk factors for the early onset LH peak.

**Table 5:** Binary logistic regression model for predicting the risk of early onset LH peak in PCOS patients

Independent variable	β	*p*	OR	95% CI lower limit	95% CI upper limit
Gn duration (days)	-0.131	0.356	0.877	0.664	1.158
Total Gn doses(U)	-0.001	0.274	0.999	0.999	1.000
Duration of infertility(years)	-0.028	0.716	0.973	0.839	1.128
BMI(kg/m^2^)	-0.009	0.860	0.991	0.893	1.099
Number of oocytes	-0.027	0.402	0.973	0.913	1.037
Basal FSH(IU/L)	0.153	0.219	1.165	0.913	1.487
Basal LH(IU/L)	0.022	0.514	1.022	0.957	1.091
Basal E2(pg/L)	-0.005	0.562	0.995	0.977	1.013
Basal P (ng/mL)	0.374	0.069	1.453	0.972	2.173
Basal T (ng/mL)	-1.066	0.103	0.344	0.096	1.240
Number of 2PN	0.034	0.420	1.034	0.953	1.122
Number of good-quality embryos	-0.117	0.113	0.889	0.769	1.028
Gn distance from antagonist days (days)	0.165	0.100	1.179	0.969	1.436
E2 on the day of hCG(pg/L)	0.000	0.132	1.000	1.000	1.001
P on the day of hCG(ng/mL)	0.256	0.157	1.292	0.906	1.844
Constant	-1.142	0.588	0.319		

Note: R2 = 0.190; F = 31.776; p = 0.007

### Relationship between LH level on the day of antagonist and basal LH level

**Fig. 2:**
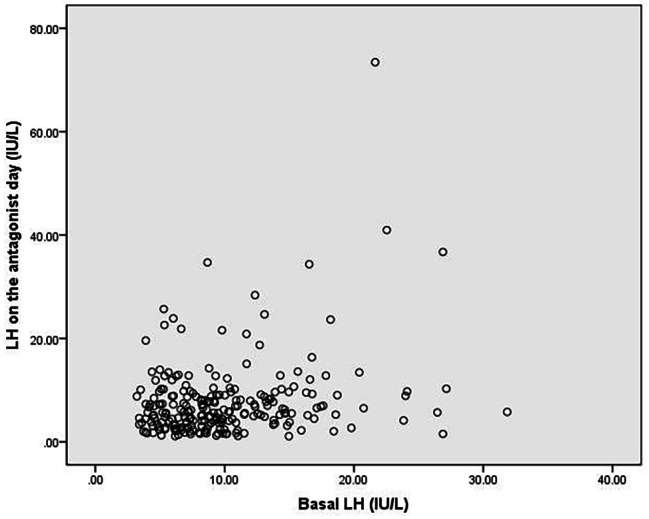
Scatter plot showing the association between basal LH levels and LH levels on the day of antagonist addition

Pearson correlation analysis showed that the basal LH levels and LH levels on the day of antagonist addition were not significantly associated (Fig. 2) (Pearson correlation coefficient = 0.259).

### The ROC curve was constructed

**Fig. 3:**
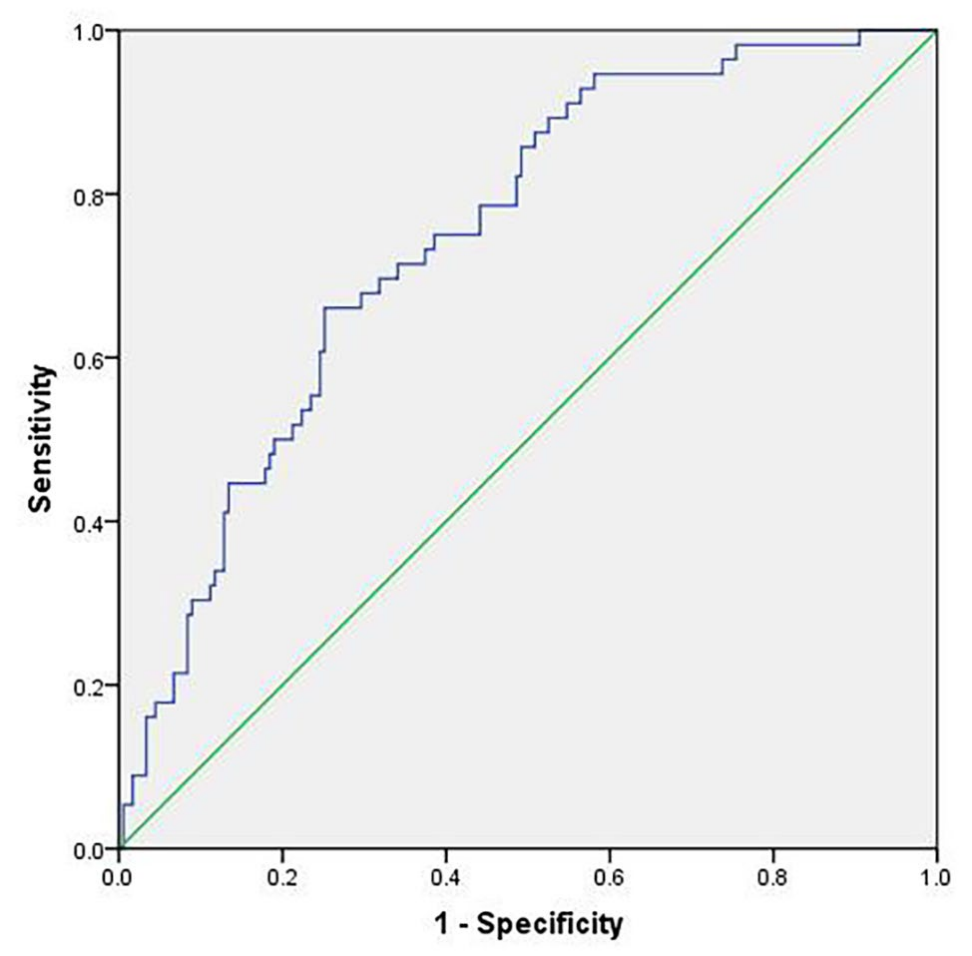
ROC curve of logistic prediction model for early onset LH peak

Using the logistic prediction model of the independent variables in Table 5 for the early onset LH peak.The AUC value was 0.747, 95%CI was 0.678–0.815,the sensitivity was 0.661, the specificity was 0.749, and the Youden index was 0.409. (Fig. 3).

## Discussion

Most scholars believe that high basal LH in PCOS patients will increase the risk of early-onset LH peak, affect the quality of oocytes, and affect the clinical pregnancy outcome. However, there is a lack of clinical studies to confirm this. In this study, we found that high basal LH did not affect the number of embryos, clinical pregnancy outcome, and the risk of OHSS. In the past, OC treatment was commonly used in clinical practice to reduce LH levels, but some scholars found that PCOS patients who had a history of OC use before the antagonist program would reduce the live birth rate and occur sempty follicle syndrome and so on directly affect the clinical pregnancy rate of IVF/ICSI [21, 22]. However, our study suggest that OC pretreatment in PCOS patients before antagonist treatment not only increases the dose of gonadotropin used during ovulation induction but also does not improve pregnancy outcomes. The analysis may be due to the lower LH level during ovulation induction, which is the reason for the increased dose of Gn used [23].

In the study shown that the number of days from Gn initiation to antagonist addition were significantly lower in the high LH group than in the low LH group. The shorter duration of Gn use may be because the higher LH levels in the early follicular phase can bind to each other and to the LH receptor on theca cells, acting as an oestrogen precursor substance (androgen). This can increase the sensitivity of early follicles to FSH, leading to rapid follicle development and shorter Gn use. Similarly, a related study also confirmed [24] that clinical pregnancy, embryo implantation, and early miscarriage rates are not significantly different between the high and low LH groups when antagonist day and hCG day are used as reference. In this study, the final clinical outcome was not significantly different between the three groups on the antagonist day and hCG day. Xiao Shan et al. [25, 26] suggested that mid follicular LH levels are closely related to ovarian responsiveness. Significant decrease or increase in LH levels during the mid follicular phase affects follicular development and endometrial receptivity, thus influencing pregnancy outcome. Luo X et al. [27] suggested that low LH levels on the hCG day decrease ongoing pregnancy and live birth rates and increase early miscarriage rates. Therefore, a reasonable LH level during ovulation induction is essential for a positive pregnancy outcome.

Therefore, the effects of LH are unpredictable, and basic and clinical evidence suggests that a luteinizing hormone stimulation threshold is required for adequate follicular development and oocyte maturation [28]. Our study showed that in women with PCOS elevated basal LH levels did not affect the outcome of IVF/ICSI cycles treated with GnRH antagonist protocols [29], and one study shown to that basal LH was not increase the risk of miscarriage in women with PCOS [30–32]. This suggests that suppression of LH values prior to IVF is of little clinical value for individualized patient management. On the other hand, endogenous LH levels are not sufficient to fully support follicle development in some patients, and this idea is being increasingly adopted worldwide.In addition, previous studies have shown that severe LH suppression is detrimental in patients receiving cycles of GnRH antagonist therapy. The role of LH in PCOS has not been fully studied and seems to be exaggerated without adequate evidence [33, 34].

So there may be other reasons why women with PCOS have lower pregnancy rates, and we know PCOS is an endocrine disorder syndrome characterized by the coexistence of reproductive dysfunction and abnormal glucose metabolism [35, 36], it have androgen hyperplasia (HA), ovulation dysfunction and polycystic ovaries are the characteristics of this syndrome [37–39]. In addition to these diagnostic features, obesity and insulin resistance (IR) are also common abnormalities associated with PCOS [40, 41]. Studies have reported that regardless of ovulation status, women with PCOS are still at risk of reduced fertility potential [42], which may be caused by pregnancy complications [43] and the combined effects of oocyte quality [44] and endometrial receptivity [45–48].

In this study, binary logistic regression analysis indicated that all the independent variables could not increase the risk of LH peak in patients. Moreover, the timing of antagonist addition did not increase the risk of early-onset LH elevation in PCOS patients (*p* > 0.05).So, antagonists should not be added too early for the PCOS population with high basal LH levels because of the cost and to avoid an increased number of injections. Herein, elevated basal LH levels did not increase the risk of an early onset LH peak. Although LH levels on the antagonist day were inversely associated with the number of good-quality embryos, correlation analysis showed that basal LH levels were not significantly correlated with LH levels on the antagonist day.The ROC curve was constructed by the logistic prediction model of the early-onset LH surge, and the results showed that the logistic joint model we constructed had a good ability to predict the early-onset LH surge.

This is a retrospective study, especially the small number of fresh embryo transfer cases included, and there may be selection bias. Although the clinical pregnancy outcomes of frozen embryo transfer were compared at the same time, the clinical conclusions have certain limitations due to the small number of cases. In the future, more large-sample and high-quality prospective studies are needed to further explore the effect of basal LH elevation on the process of ovulation induction in PCOS patients, in order to improve the pregnancy outcomes of PCOS patients in assisted reproduction. However, according to the results of this study, from the data analyzed, high basal LH level does not affect the outcome of IVF/ICSI in PCOS patients, so it is not recommended for PCOS patients to undergo pretreatment before entering the antagonist program.

Our team future research direction: on the one hand, the relationship between basal LH and antagonist day LH on normal ovarian response and low ovarian response related cases and the influence of assisted pregnancy outcome were further studied, and the influence of LH on oocytes, embryo quality and embryonic development potential was also paid attention to and further studied. On the other hand, studies focused on weight and ages in relation to oocyte quality and live birth rate in pcos patients.

## Data Availability

The data that support the findings of this study are available on request (sdwangkh@126.com).

## Abbreviations

BMI: Body Mass Index
FSH: Follicle-Stimulating Hormone
LH: Luteinizing Hormone
E2: Estradiol
P: Progesterone
T: Testosterone
2PN: 2pronucleus
hCG: Human Chorionic Gonadotropin
Gn: Gonnadotrophin
IVF: In-vitro fertilization
ICSI: Intracytoplasmic sperm injection
OHSS: Ovarian Hyper-stimulation Syndrome
PCOS: Polycystic ovarian syndrome
GnRH: Gonadotropin-releasing hormone
OC: Oral contraceptives
COH: Controlled ovarian hyperstimulation
AFC: Antral Follicle Coun
